# Effectiveness of oncogenetics training on general practitioners' consultation skills: a randomized controlled trial

**DOI:** 10.1038/gim.2013.69

**Published:** 2013-05-30

**Authors:** Elisa J.F. Houwink, Arno M.M. Muijtjens, Sarah R. van Teeffelen, Lidewij Henneman, Jan Joost Rethans, Liesbeth E.J. van der Jagt, Scheltus J. van Luijk, Geert Jan Dinant, Cees van der Vleuten, Martina C. Cornel

**Affiliations:** 1 Department of Clinical Genetics, Section of Community Genetics, EMGO Institute for Health and Care Research, VU University Medical Center, Amsterdam, The Netherlands; 2 Department of General Practice, School for Public Health and Primary Care, Maastricht University, Maastricht, The Netherlands; 3 Department of Educational Development and Research, Faculty of Health, Medicine and Life Sciences, Maastricht University, Maastricht, The Netherlands; 4 Skills Lab, Faculty of Health, Medicine and Life Sciences, Maastricht University, Maastricht, The Netherlands; 5 The Dutch College of General Practitioners (NHG), Utrecht, The Netherlands; 6 Department of Education and Resident Training, Maastricht University Medical Centre, Maastricht, The Netherlands

**Keywords:** general practice, genetic consultation skills, genetics education, hereditary cancer, oncogenetics, standardized patients, training

## Abstract

**Purpose:**

General practitioners are increasingly called upon to deliver genetic services and could play a key role in translating potentially life-saving advancements in oncogenetic technologies to patient care. If general practitioners are to make an effective contribution in this area, their genetics competencies need to be upgraded. The aim of this study was to investigate whether oncogenetics training for general practitioners improves their genetic consultation skills.

**Methods:**

In this pragmatic, blinded, randomized controlled trial, the intervention consisted of a 4-h training (December 2011 and April 2012), covering oncogenetic consultation skills (family history, familial risk assessment, and efficient referral), attitude (medical ethical issues), and clinical knowledge required in primary-care consultations. Outcomes were measured using observation checklists by unannounced standardized patients and self-reported questionnaires.

**Results:**

Of 88 randomized general practitioners who initially agreed to participate, 56 completed all measurements. Key consultation skills significantly and substantially improved; regression coefficients after intervention were equivalent to 0.34 and 0.28 at 3-month follow-up, indicating a moderate effect size. Satisfaction and perceived applicability of newly learned skills were highly scored.

**Conclusion:**

The general practitioner–specific training proved to be a feasible, satisfactory, and clinically applicable method to improve oncogenetics consultation skills and could be used as an educational framework to inform future training activities with the ultimate aim of improving medical care.

Genomics holds great promise to improve human health. Genetics of common disorders (diabetes, cancer, cardiovascular diseases) and monogenic subtypes (maturity-onset diabetes of the young, *BRCA1/2*, familial hypercholesterolemia, and long QT syndrome), in particular, are expected to come increasingly to the forefront in primary care. Consequently, general practitioners (GPs) are facing a daunting informational challenge to keep abreast of the expanding body of genomics knowledge and attain competencies for informed use of its potential for personalized patient care.^1^ In view of increasing requests for DNA-based predictive testing arising from a positive family history and GPs' increasing involvement in preventive checkups, it is important for GPs to be competent to take and interpret a family history and deal appropriately with patients' questions and concerns.^2^ Each family practice has a substantial number of unidentified asymptomatic patients with relatively young first-degree relatives with familial or hereditary forms of cancer (breast, ovarian, uterine, and colorectal cancer), and such patients should be referred to a clinical geneticist for counseling and/or screening according to guidelines.^3,4^ Women carrying a *BRCA1/2* mutation, for example, have a lifetime risk of 60–80% of developing breast cancer (accounting for 5–10% of all breast cancer cases), and timely identification enables them to benefit from otherwise unexploited life-saving “risk-management options,” such as salpingo-oophorectomy and/or mastectomy, annual screening, and pharmaceutical chemopreventive options.^5^ Assessing familial risk by taking a family history can be a reliable method to improve outcomes of hereditary forms of cancer with targeted cancer prevention strategies.^6,7,8,9^ Taking an adequate family history, however, is difficult and time consuming. Insufficient genetics knowledge and consultation skills to actually conduct an initial oncogenetics risk assessment and its interpretation pose a barrier to appropriately recognize and elicit details to assess the features of potential oncogenetic risks.^6^ This could warrant timely referral to oncogenetics services for further assessment and genetic testing (referral-level competences). Moreover, lack of computerized decision support implies that GPs themselves need to learn how to adequately interpret family history and act on it.^10^ Educational innovation therefore seems imperative, including genetic risk ascertainment and prevention.^11^ Unless GPs receive proper education and training, individual genetic care by GPs will likely be unhelpful and possibly even harmful.^3,12,13^ Considering the urgent need for and the potentially huge benefits to be gained from genetics education for GPs, we embarked on an educational project aimed at strengthening the role of genetics in family medicine.

Well-defined core genetics competencies for nongenetic health-care workers are considered a precondition for the development of effective genetics education.^14,15^ Educational activities should be responsive to GPs' assessed needs with respect to cognitive (knowledge), psychomotor (consultation skills), and affective (attitude) aspects of genetics competence. Previous studies have shown that as far as genetics is concerned, nongenetic health-care workers require not only education but also clear guidelines and definitions of their responsibilities.^16,17^

This article reports a study in which GPs attended a needs-based, interactive oncogenetics training aimed at enhancing their insight, consultation skills, and attitudes relevant to the identification of oncogenetic disease in family practice consultations. We evaluated the effects of the training in two ways: (i) office visits by standardized patients (SPs) to determine the extent to which GPs synthesized and applied the newly learned behaviors and (ii) questionnaires to determine GPs' satisfaction with the training and perceived applicability of the new genetics consultation skills in their practice.

## Materials and Methods

### Experimental design

We conducted a pragmatic, blinded, randomized controlled trial (RCT) with parallel repeated measurements using a performance checklist and questionnaires. Kirkpatrick's four-level framework for evaluating educational outcomes entails (i) valuation (satisfaction), (ii) learning (knowledge and knowledge retention), (iii) behavior (applying knowledge about timely identification of patients at risk and referral), and (iv) effects on patient health and organization (change in practice and results).^18,19^ The design included an innovative measurement method with office visits by SPs aimed at the third level. Unannounced SP visits are a proven method to collect data about real practice in a direct and reliable way.^20^

Participating GPs were randomly assigned to a training date: December/January for the intervention group (four sessions) or March/April for the control group (three sessions). The trial ran from December 2011 to April 2012.

The RCT involved an intervention (oncogenetics training) and repeated measurements before the intervention (T0), 1 month after the intervention (T1), and 3 months (T2) after the intervention (**Table 1**). All participants completed a demographics survey at T0. Between T0 and T1, the intervention group attended the training, whereas the control group received no intervention. For the evaluation of genetic consultation and of consultation skills at T0 (pretest), T1 (posttest), and T2 (retention test), SPs were asked to complete checklists after consultations with both GP groups (level 4).^21^ The SPs were blinded to the GPs' group assignment. To measure satisfaction with the training (level 1), the intervention group completed a questionnaire at T1. To measure the participants' perceived applicability of the training content (level x), the intervention group answered a questionnaire at T2. To stimulate compliance of control group participants, they were invited to attend the training after T2.

The ethical review boards of the Netherlands Association for Medical Education, Maastricht University Medical Center, and VU University Medical Center, Amsterdam, The Netherlands, approved the study protocol. All participants gave written informed consent before the trial.

### Participants

The project team collaborated with the Dutch College of General Practitioners (NHG) and local training providers to recruit GPs working full or part time in general practice.

For logistic reasons, recruitment was limited to all GPs practicing in two Dutch provinces, who received an invitational online mailing with information about the aim of the study, the contents of the face-to-face training, and the evaluation procedure. Accreditation points were offered to GPs completing the study. A book on genetics in general practice or a book voucher of equal value was offered as an extra incentive. Four e-mail or telephone reminders were sent to nonresponders.

**Figure 1:**
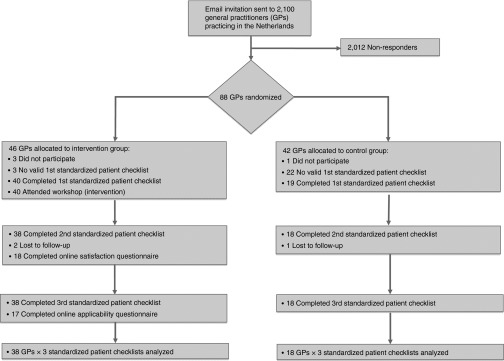
**Consort diagram of randomization and participation.**

Eighty participants were needed to detect a medium to large sized effect with a power of 90% and significance level of 5%.^22^ **Figure 1** shows the randomization scheme and participation.

### Intervention

The intervention group attended a 4-h face-to-face evening training covering oncogenetic consultation skills (family history, familial risk assessment, and efficient referral), attitude (medical ethical issues), and clinical knowledge required in primary-care genetic consultations. More specifically, the training comprised the following educational content aimed at equipping GPs to:

- Recall clinically relevant information about types of hereditary cancer (breast, ovarian, colon, skin), including genes associated with oncogenetics syndromes most commonly tested for;
- Recognize patients with features suggesting inherited predisposition to cancer;
- Draw a family tree as a tool to identify patients at risk;
- Discuss (possible) familial and hereditary cancer risks, management of potentially developing hereditary cancer (i.e., surveillance and risk-reducing surgical options), and related ethical issues;
- Identify patients for referral for risk assessment and find relevant information online using oncogenetics guidelines;
- Explain the possibilities and limitations of oncogenetic testing; and
- Know when to consult and/or refer to a genetics specialist.

The training was developed by a multidisciplinary team consisting of an NHG educational expert (L.E.J.v.d.J.), a GP researcher (E.J.F.H.), two clinical geneticists, and two educationalists (S.J.v.L and C.v.d.V.). The focus was on oncogenetic diseases with relatively high prevalence in family practice (breast cancer due to *BRCA* mutations, colon cancer (e.g., familial adenomatous polyposis, Lynch syndrome) due to *APC*/mismatch–repair gene mutations, and skin cancer (e.g., familial atypical multiple mole melanoma syndrome due to *CDKN2A* (p16) gene mutations)). The training started with a 1-h interactive theoretical session on hereditary forms of cancer led by a clinical geneticist from a local academic hospital who was familiar with the aims of the program, followed by a 1-h session with two patients of the Dutch BRCA patient organization, who talked about their experiences, discussed ethical issues, and answered questions. A short break was followed by a 2-h workshop in which participants in small groups engaged in three role-playing consultations for three oncogenetic problems in the presence of experts (clinical geneticist, patient representatives, and two trainers). Patients and GPs were role-played by participants, and the others gave feedback.

### Measurements

*Standardized patients.* For a detailed description of the training sessions with SPs preceding the practice visits, clinical case scenarios, and development and finalization of the checklist, see **Supplementary Tables S1 and S2** online; **Supplementary Materials and Methods** online.^23^

*Questionnaires.* Three online self-reported questionnaires were used to collect data on satisfaction, applicability of new consultation skills, and demographics and practice characteristics (see **Supplementary Tables S3, S4,** and **S5** online). The instruments were developed and validated in collaboration with the research team. The questionnaires were developed and validated in collaboration with content experts (experts in daily clinical genetics, a GP, and an expert in education and questionnaire development) and pilot tested.

The satisfaction questionnaire contained two items with five-point Likert scales (1: completely disagree; 5: completely agree) (In the questionnaires, the coding was directed oppositely (1: completely agree, 5: completely disagree) in accordance with the conventions of the NHG. For ease of interpretation, in the current article, the ratings were recoded to comply with international conventions (1: completely disagree, 5: completely agree).) and an item with a global rating on a 10-point scale. The applicability questionnaire contained six items with five-point Likert scales and one item with a four-point ordinal scale.

### Regression analysis

For a detailed description of regression analysis to investigate improvement of genetic consultation behavioral skills, see **Supplementary Materials and Methods** online.

Satisfaction with the intervention and applicability scores was analyzed by calculating the mean scores, 95% confidence intervals, and SDs for the pooled data from the satisfaction questionnaire. All analyses were performed using SPSS version 19 (SPSS, Chicago, IL).

## Results

### Randomization and dropout comparisons

Of 88 randomized GPs who agreed to participate in the training in December/January 2011/2012 (intervention group) or March/April 2012 (control group), 56 (38 intervention, 18 control group) completed the entire procedure, and 32 were lost to follow-up due to lack of time or sickness (**Figure 1**).

### Participant characteristics

Participants in the intervention and control groups did not differ significantly in age, sex, years of experience, type of office, or office situation (**Supplementary Table S6** online).

### Effects of the intervention on oncogenetics-related learned consultation skills

Each of the 56 family physicians was visited by three SPs, portraying different cases, resulting in 168 first visits (**Figure 1**).

**Figure 2:**
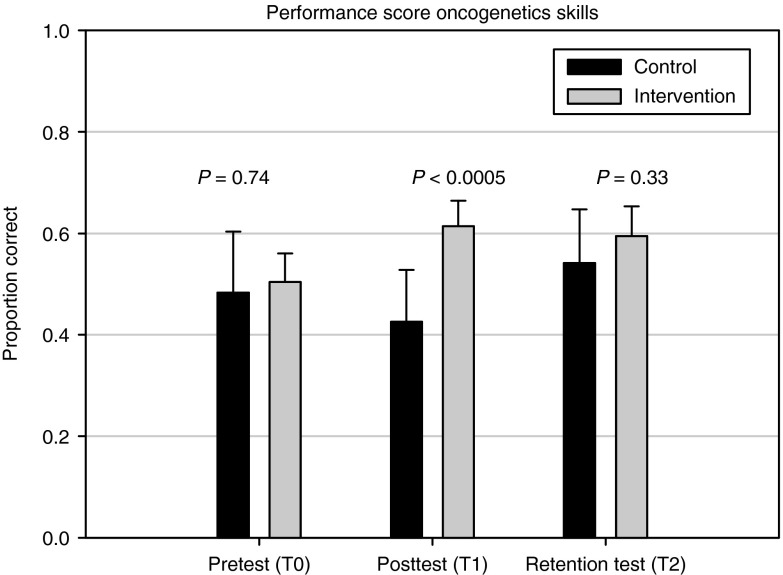
**Performance scores for oncogenetics consultation skills as measured by proportion correct on SP checklists (mean and 95% confidence interval) for control group (black) and intervention group (gray) at T0, T1, and T2, corresponding to pretest, posttest, and retention test measurements, respectively.** SP, standardized patient.

**Figure 2** shows the raw mean performance scores (proportion correct) for the control and intervention groups at times T0 (pretest), T1 (posttest), and T2 (retention test). Between-group differences were found to be nonsignificant for the pretest and retention test, but the posttest difference of 0.19 in favor of the intervention group was found to be significant (*t*-test; *P* < 0.0005). These estimations, however, are based on raw means and may be biased due to differences in difficulty between the three SP cases. More precise and unbiased estimations were obtained by the regression analysis (**Table 2**). The regression results for the T1 score showed that the effect of the intervention (the coefficient for training) was statistically significant and amounted to 0.14 on the proportion-correct scale; the corresponding value for the standardized regression coefficient was equal to 0.34, indicating a moderate effect size. The analysis for the T2 score showed that the significant intervention effect persisted until the retention measurement at T2 (2 months later) and amounted to 0.11 (standardized regression coefficient = 0.28, moderate effect size). Hence, the performance improvement due to the intervention was still substantial at T2, being equal to 80% of the immediate effect at T1.

Effect modification of the treatment effect by baseline value was tested for the T1 and T2 scores; for both variables, the effect modification was found to be nonsignificant.

### Satisfaction and applicability

The satisfaction questionnaire resulted in high scores for the two items (both 4.4) and a global score of 7.7; when applicability is also considered, favorable scores were found for all six items (3.5–4.5). Overall, 65% of the trainees reported applying the newly learned skills monthly, and 35% weekly (**Table 3**).

## Discussion

### Summary of main findings

To our knowledge, this is the first RCT to use SPs to investigate improvement of GPs' oncogenetic professional behavior after attendance of an oncogenetics training. The results show sustained improvement 3 months after the training, as well as high satisfaction with the training and positive perceptions of the practical applicability of the training topics.

Immediate and long-term training effects were evaluated at Kirkpatrick's level 3 (behavior showing evidence of learning), which enhances the value of the findings.^22,24^ The results indicate that case-based oncogenetics education can achieve sustained improvement with a moderate effect size in urgently desired genetics competencies for GPs, whereas the positive results for satisfaction and applicability may reflect a move toward a culture of genetic medical practice improvement. Educational interventions likely have a small-to-moderate effect on physician knowledge and performance, and patient outcomes.^25^ A few factors that were applied could have supported this result, such as active and interactive sessions, and single-group and smaller-group sessions. Whether there is a sustainable impact on applicability of the training in practice, including timely identification of patients with a possible cancer predisposition syndrome and appropriate referral, will need further longer-term studies. Designed to fill gaps in physicians' competencies and boost their confidence in using basic clinical genetic principles and activities,^26,27,28,29,30^ the oncogenetics training addressed previously prioritized key features of genetic consultation skills and attitude but not basic science knowledge.

In previous studies, SPs have been used successfully to assess changes in clinical competence and performance and sustained effectiveness of behavioral training^31,32^ but not to evaluate attitudinal factors cited as directing practice performance, such as patient satisfaction.^21^

After the training, the participating GPs seemed to be more comfortable incorporating oncogenetics aspects into patient consultation skills, as reflected in their high perceived applicability. It seems plausible that this, in turn, will enhance efficient and effective referral for genetic counseling. Whether the latter effect will materialize, however, remains to be examined in future studies. Taylor et al.^33^ discussed barriers to effective primary-care involvement in the expanding field of adult genetics, arguing that genetic medicine should be part of integrated medical care and therefore of primary-care medicine. We agree with this viewpoint and feel that the training we designed shows promise to enhance communication between GPs and the genetics community, identification of high-risk patients, and timely referral to genetics services.

### Methodological considerations

One of the aims of including real patients and simulated consultations in our training was to promote a favorable attitude among GPs toward the application of genetic competencies. A study by Carroll et al.^34^ measured intent to use clinical genetics scenarios and increase competence due to a multifaceted knowledge translation intervention but used questionnaires and not ratings of observed practice behaviors. Patient and societal perspectives on legal consequences of DNA-based testing results (e.g., being able to find a genetics information source or ability to obtain a mortgage or life insurance), however, demand that physicians' effective use of genetics be demonstrated by actual performance in health-care practice.^7,21^ We therefore deliberately deployed trained and blinded SPs to optimize the value of the measurement. Repeated SP visits may have impacted the outcome of learning effects in both study groups, because the GPs would have had a higher level of awareness of being critiqued and could have felt a certain pressure to perform appropriately, but this is controlled for in the current study thanks to its RCT design.^35^

Rollnick et al.^26^ suggested that learner-directed and context-bound consultation skills training should be integrated into everyday practice in a way that is acceptable to clinicians. On the basis of this principle, we had physicians identify their training needs and tailored our training to the practice context by patient-centered consultation skills training. On the basis of the results of our earlier studies, we emphasized everyday genetic clinical experiences more than consultation skills and attitude alone.^16,17^ Our strategy could therefore be described as an “enriched context-bound consultation skills training.” Informal comments after the training by participants made clear that this format had a positive effect on their learning.

Potential oncogenetic problems are considered very personal topics to discuss between a patient and their GP.^16^ This is why it was not discussed in an incognito setting with a so-called “new” patient or unannounced, concealed simulated patient.^27^

### Strengths and limitations

A strength of the study was measuring change in consultation skills after the training by using SPs in particular, as opposed to using computerized case-based testing, for example. A variation of measurement instruments was proposed to predict practice performance.^28^ SP-based measurement is relatively unobtrusive, highly authentic, and based on patient perception. Another strength of the study is the fact that the educational intervention was tailored to the learners' needs.^6,16^ Because the current study is confined to one health-care setting within one country, the generalizability of the results may be limited. The training's demands on resources, facilities, and logistics may limit the feasibility of training delivery in many different settings. Nevertheless, SP-based assessment is a valid instrument to describe what happens in real practice and can therefore provide valuable information for advanced development of genetic trainings. The study design introduces the possibility of bias by virtue of each GP seeing three different case presentations in different orders. This potential limitation was acknowledged, and statistical accommodations were made.

Using comparable case scenarios in this study, it was possible to measure change in checklist scores over time. However, it remains to be investigated whether it is possible to use different scenarios, for example, based upon on a family history alone. This would be a scenario seen in daily GP practice and the time when timely referral could be of benefit to the presymptomatic patient in regards to preventing or reducing familial cancer risk. Future studies could include assessing the issues addressed in the study by Culver et al.,^29^ namely, satisfaction with the time to address concerns, acknowledgments of patient concerns about cancer risk by physician, and offering reassurance. Using the validated checklist, the current study measured GPs' genetics consultation skills, thereby reflecting that training outcomes' covering the full scope of good practice consultations: key ingredients related to family history taking, genetic risk assessment, and referral to genetics specialists. The SPs requested standard 10-min appointments. This may seem short for a first consultation; however, this is a standard duration in the Netherlands. If requested, in “real-world” clinical practice, a follow-up appointment could be made to adequately address all the concerned issues. However, in the study design, the SPs came in with a concern possibly related to an inherited form of cancer. The extent to which GPs synthesized and applied the newly learned behaviors was assessed by long-term changes in a 28-item checklist score, not by whether all issues would be discussed. Performance assessment is considered representative of a product of competence, influences of individual (e.g., health, relationships), and organization (e.g., facilities, practice time). GPs were therefore similarly assessed for performance under equivalent conditions (e.g., appointment time limitation focused entirely on the sole reason for the visit, without distraction or delay).^21,30,32^

Although the 3-month study period may have been too short to detect sustainable practice improvement in the long term, repeated measurement of consultation skills predicts practice performance in the long term.^21,31^

Voluntary participation by interested GPs could have caused selection bias. However, similarity of the baseline characteristics of the two groups and comparability of the 60% participation rate with that of other studies among GPs^36,37^ indicate that the participants were representative of GPs likely to attend oncogenetic training in the future. Furthermore, it is possible that participating in the oncogenetics training might become part of standard training for all GPs. There was an imbalance however between the dropout rate in the intervention and control group and the reason for this is not clear. Attending the training in the beginning of the trial period could have provided the urgent information to be able to satisfactorily finish all measurements long-term. Participants in the control group on the other hand had to wait for training content, possibly causing resistance to finish all measurements resulting in dropout. General reasons for dropout were reported (no time and sickness) but not specific attributes. It is therefore unlikely that self-selection in dropout negatively impacted the validity of the results.

A pragmatic and blinded study design has known limitations.^38^ Obviously, it is preferable for an RCT in which participants are blinded to inclusion in the intervention or control group, and those conducting the measurements are blinded as well.^39^ We achieved this by blinding the SPs to the GPs' group allocation and by having two independent researchers (A.M.M.M. and S.R.v.T.) analyze the checklist scores in a blinded manner.

The results indicate that an oncogenetics training designed to meet GPs' educational needs can be a satisfactory and feasible method for sustained improvement of competencies to ensure appropriate application in family medicine of developments from the rapidly evolving field of genetics. Learner-directed and context-bound genetics education appears to be a valuable tool to stimulate GPs to deliver genetic services.^40^ We plan to use the results to inform the design of new trainings on complex genetic diseases, including hereditary forms of cancer, cardiovascular disease, and diabetes, in our continuing efforts to improve referral strategies and timely recognition of high-risk genetic patients. Large-scale international RCTs with adequate power are warranted to further assess how genetics education can improve health care.

## Disclosure

The authors declare no conflict of interests.

## Figures and Tables

**Table 1:**
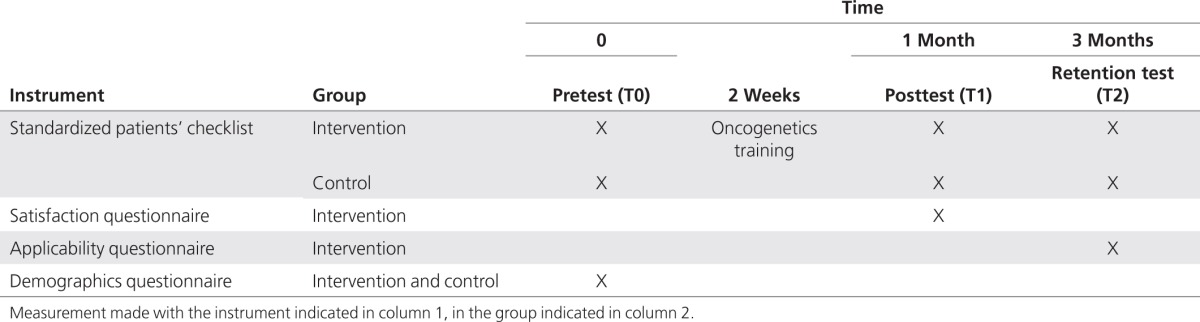
Timetable of the randomized controlled trial showing scheduled measurement times, instruments, and measurements made (indicated with X) in the intervention and control groups

**Table 2:**
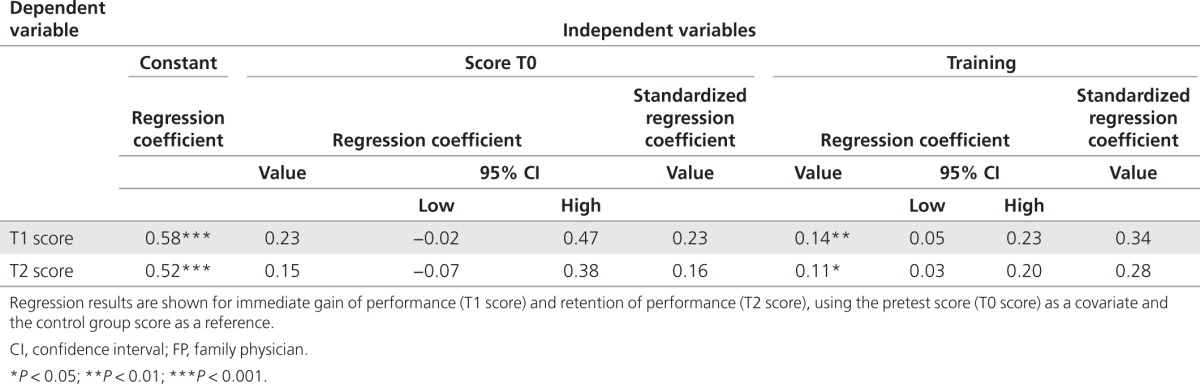
Effect of the oncogenetics training on the performance of GPs

**Table 3:**
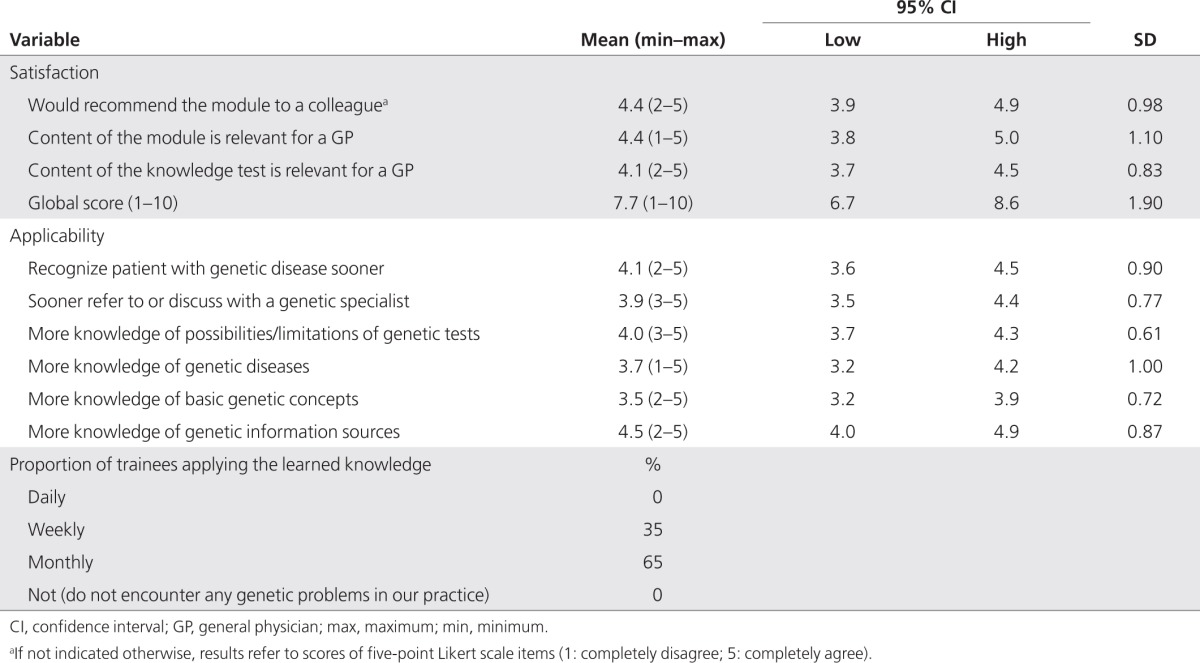
Satisfaction (intervention only; *n* = 18) and self-reported applicability (intervention only; *n* = 17) as a result of oncogenetics training
