# Supplementary material for: Effectiveness of oncogenetics training on general practitioners' consultation skills: a randomized controlled trial
Source: Genet Med. 2013 May 30;16(1):45–52. doi: 10.1038/gim.2013.69 (PMC3914027; doi:10.1038/gim.2013.69)
Supplement: Supplementary Table S1 [file gim201369x1.doc]

| **Table S1. Description of Cases presented by Standardized Patients** | | | |
| --- | --- | --- | --- |
|  | **Skin cancer case** | **Colon cancer case** | **Breast cancer case** |
| **Biography** | Male or female, age 31. Born and raised in suburb of major city. Went to culinary school, works at restaurant. Lives in apartment downtown, romantic relationship ended two years ago. | Male or female, age 41. Born in small city, moved to outskirts 10 years ago. After high school, first worked in do-it- yourself store, past 15 years as taxi driver. Relationship with spouse is stable. | Female, age 41.  Lives in quiet suburb with husband, daughter age 13 and son age 10. After college began working at bank. Family situation is stable, spends a lot of time with children in the weekend. |
| **Medical complaint** | Spots on back which bleed, itch and are getting bigger. Last month doctor called it a regular birthmark. Spots noticeable for at least 3 months. | Pain in lower left abdomen and watery brown bowel movements. Symptoms began 3 weeks ago after trip to Thailand. Obesity, but recently lost 1 kilo. | Lump in left breast, noticed last week. The 4 cm irregular swelling not painful but sensitive. The skin on the swelling is a little red and dimpled. |
| **Lifestyle** | Smokes half a pack of shag a day since age 17. Drinks heavily twice a week after work. No drugs, no coffee, tea once a day. Sunbathing vacation in southern France every year. | No history of smoking. Drinks wine occasionally. On Mirtazipine (anti-depressant) for a year. Drinks 4 cups of coffee and 2 cans energy drink per day. Fresh vegetable is absent from diet, does eat 2 fruit servings. | Heavy smoker from age 16 up to 2 years ago. Drinks wine with dinner and social drinker in the weekend. Drink 2 cups of coffee a day. |
| **Medical history** | No medical history.  Family history:   - Sister: skin cancer at age 23, died two years later of brain tumor. - Father: skin cancer at age 40, treated and cured. - Grandfather, father’s father: skin cancer at age 43, died 5 years later of brain tumor. - On mother’s side no one has cancer. | - Mild depression - Obesity;BMI 35   Family history:   - Father diagnosed with colon cancer at age 40, died of it a year later. - No contact with rest of family. | No medical history.  Family history:   - Grandfather, mother’s father: unknown cancer and died, age 55. - Mother: breast cancer at age 35 and is now 75. - Sister: ovarian cancer at age 30 and died at age 33; - other 2 sisters seem healthy. - On father’s side no one has cancer. |
| **Motivation for appointment** | You want a thorough examination, because you know how quickly cancer can develop. If the doctor’s diagnosis isn’t cancer, you will need convincing. You know that your sister had the same symptoms back then and it took a lot of persistence to get a referral and diagnosis. | You want a solution to your symptoms, and clarity on whether it’s cancer or not. You know your father got cancer at your age and it killed him. You don’t mention this until later; you don’t like to talk about family. You don’t have much contact with relatives due to conflicts. | You’re scared of cancer because there’s a lot of it in the family. You want to know whether the lump will get larger and if it’s malignant. You really want to be referred to the specialist. If the doctor doesn’t do this, let him or her know you’re really scared and be disappointed. |
| **Physical examination results**  **(given in paper format by the SP to the FP during the visit if requested)** | *Inspection:* A lesion is visible on the right shoulder blade. The lesion has an erratic surface, irregular border and color, and is 2 cm in diameter. | *Inspection:*  no irregularities. *Auscultation:* plumbing sounds. *Percussion:* normal sounds in all four quadrants of abdomen.  *Palpation:* no palpable irregularities. Rectal exam: normal. | *Inspection:* no irregularities. *Palpation:* a palpable mass in the left breast. Size: 2cm. Consistency: solid-elastic. Irregular shape. Not painful, no inflammation. No discharge from nipple. |
