# Supplementary material for: Effectiveness of oncogenetics training on general practitioners' consultation skills: a randomized controlled trial
Source: Genet Med. 2013 May 30;16(1):45–52. doi: 10.1038/gim.2013.69 (PMC3914027; doi:10.1038/gim.2013.69)
Supplement: Supplementary Table S2 [file gim201369x2.doc]

| **Table S2.** Standardized patient checklist items assessing genetic consultation skills | | | | |
| --- | --- | --- | --- | --- |
|  | Score as yes: | **YES** | **NO** | **?** |
| 1. Responds to my concern about possible genetic cancer. | “I can imagine you are worried about that”, “what are you concerned about?”, etc. |  |  |  |
| 2. After expressing my concern, the GP confirms that this form of cancer can be hereditary. | “This form of cancer can be hereditary”, “this form of cancer is often passed in families”, etc. |  |  |  |
| 3. Asks about cancer in my family. | “Do any of your relatives have cancer?”, etc. |  |  |  |
| 4. Further asks which kinds of cancer occur in my family. | “Which kind of cancer did they have?”, “Can you remember what kinds of cancer your relatives have?”, etc. |  |  |  |
| 5. And asks at which age the cancer was diagnosed. | “At which age did the doctors discover that your relative had cancer?”, “How old were they when they got the diagnosis?”, etc. |  |  |  |
| 6. Asks who was diagnosed. | “Can you remember who had this kind of cancer?”, “Which relatives have this kind of cancer?” |  |  |  |
| 7. Asks specifically if this kind of cancer is present in my children, siblings or parents (first -degree relatives). | “Have your children, parents or siblings had this kind of cancer?” |  |  |  |
| 8. Draws a family tree to clarify possible inheritance of cancer. | A family tree is drawn. |  |  |  |
| 9. Explains the possibilities and limits of genetic testing. | “What we can/can’t determine by genetic testing is…”, “With genetic testing we can diagnose X but not Y.” |  |  |  |
| 10. Explains that a genetic test for this kind of cancer does not prevent that I may or may not get that cancer in the future. | Makes it clear that interpretation of tests is still limited. “This test doesn’t offer certainty that you will or won’t get this form of cancer at some point in your life.” |  |  |  |
| 11. Explains what the consequences could be for me if a certain genetic test for hereditary cancer comes back positive. | “If the test indicates that you have this inherited defect, then…”, “A positive test result would mean for you…” |  |  |  |
| 12. Asks about my expectations regarding the possibilities and limits of genetic testing. | “what do you expect from the genetic test?”, “Do you expect to have certainty after having the test?” |  |  |  |
| 13. Explains why referral to the clinical geneticist is or is not useful. | “Referral to a clinincal geneticist can be useful in your case”, “a referral in your case isn’t useful because…” |  |  |  |
| 14. Considers looking up referral criteria on the internet (possibly with me) | “I’ll look online if there is an indication to refer you to the clinical geneticist”, “to be sure that it’s useful to refer you to the clinical geneticist, I’ll look up their website” |  |  |  |
| 15. Knows about relevant clinincal geneticists in the region (possibly by looking with me online) | “By this department of clinincal genetics are the following possibilities…”, “Let’s together look at what the clinical geneticists can do for you.” |  |  |  |
| Table continued |  |  |  |  |
| 16. Considers contacting (by phone) the clinical geneticist to discuss possible referral (possibly with me) | “I will contact the specialist in genetics to discuss your situation”, “I’m calling the clinical geneticist to be sure we make the right decision” |  |  |  |
| 17. Explains when I would meet the criteria for referral to clinical genetics | “The following criteria must be met for a referral …”, “If this happens, then I can refer you to clinincal geneticist” |  |  |  |
| 18. Discusses with me whether relatives may be informed that referral is useful for them | “Perhaps it’s a good idea to inform your relatives and have them tested as well”, “It’s important that your family members also get tested for this condition” |  |  |  |
| 19. Discusses with me whether relatives should make a GP appointment, if it turns out it is hereditary cancer | “Your relatives who also come to this practice, could also have a higher risk and should be tested”, “Because your family members could have the same condition, I would like to also ask them to have a genetic test.” |  |  |  |
| 20. If yes, discusses with me whether relatives may receive information about me. | “Do you have a problem with that?“, “What do you think about that?” |  |  |  |
| 21. Indicates that cancer indeed occurs more often in my family than one would expect | “cancer indeed occurs more often in your family than I would expect”, “Your family does have a lot of this kind of cancer” |  |  |  |
| 22. Indicates that this could point to a hereditary form of cancer. | “We do see this kind of cancer in hereditary form”, “It’s possible that this kind of cancer is inherited” |  |  |  |
| 23. Registers the genetic risk (family history) of this kind of cancer in the computer system | “I’m registering this genetic risk of this kind of cancer in your record”, “I see you typing, what are you entering, is it in my record?” |  |  |  |
| 24. Mentions possible consequences of the genetic risk for my children, cousins/nieces and nephews | “The chance that your children get this condition is…”, “There’s a higher chance that your nieces will develop this condition” |  |  |  |
| 25. Refers me to the closest clinical genetics outpatient clinic | “I would like to send you to the clinical genetics outpatient clinic”, “They can help you better at the clinical genetics outpatient clinic, so I’m sending you there.” |  |  |  |
| 26. When I ask for more information to read at home, the doctor mentions [www.erfelijkheid.nl](http://www.erfelijkheid.nl/) (a Dutch website on general genetics topics) or another website | “If you want to read more info on the internet at home, you can look at [www.erfelijkheid.nl](http://www.erfelijkheid.nl/) “, “At (another site) you can find more info” |  |  |  |
| 27. Gives me a patient information letter | “In this letter you can read more information” |  |  |  |
| 28. Mentions possible support from the relevant patient organisation when I ask where else I can turn to with my concerns | “You could find support by a patient organisation”, “A specific patient group could support you with your condition” |  |  |  |
