# Supplementary material for: Effectiveness of oncogenetics training on general practitioners' consultation skills: a randomized controlled trial
Source: Genet Med. 2013 May 30;16(1):45–52. doi: 10.1038/gim.2013.69 (PMC3914027; doi:10.1038/gim.2013.69)
Supplement: Supplementary Table S3 [file gim201369x3.doc]

| **Table S3. Satisfaction Questionnaire** |
| --- |
| 1. I would recommend this training to my colleagues.  (On a scale of 1=Totally Agree to 5=Totally Disagree, 6= Not applicable/ No opinion.) |
| 2. In general, I judge the topics presented in the training as relevant for family practice.  (On a scale of 1=Totally Agree to 5=Totally Disagree, 6= Not applicable/ No opinion.) |
| 3. Which grade would you give this training, on a scale of 1= Bad to 10= Perfect? |
