# Supplementary material for: Effectiveness of oncogenetics training on general practitioners' consultation skills: a randomized controlled trial
Source: Genet Med. 2013 May 30;16(1):45–52. doi: 10.1038/gim.2013.69 (PMC3914027; doi:10.1038/gim.2013.69)
Supplement: Supplementary Table S4 [file gim201369x4.doc]

| **Table S4. Perceived Applicability Questionnaire** |
| --- |
| 1. I apply the knowledge gained from this training:   - Daily - Weekly - Monthly - I don’t come across any genetic issues in my practice and therefore do not apply the knowledge from the training. |
| 2. I recognize patients with a genetic condition much earlier than I did before I completed the training.  (On a scale of 1=Totally Agree to 5=Totally Disagree, 6= Not applicable/ No opinion.) |
| 3. I refer to or consult with a clinical geneticist much earlier than I did before I completed the training.  (On a scale of 1=Totally Agree to 5=Totally Disagree, 6= Not applicable/ No opinion.) |
| 4. I have more knowledge about the possibilities and limits of genetic testing than I had before I completed the training.  (On a scale of 1=Totally Agree to 5=Totally Disagree, 6= Not applicable/ No opinion.) |
| 5. I have more knowledge about the most common genetics conditions in the Netherlands than I had before I completed the training.  (On a scale of 1=Totally Agree to 5=Totally Disagree, 6= Not applicable/ No opinion.) |
| 6. I have more knowledge about fundamental concepts of genetics than I had before I completed the training.  (On a scale of 1=Totally Agree to 5=Totally Disagree, 6= Not applicable/ No opinion.) |
| 7. I have more knowledge about important sources of information about genetics than I had before I completed the training.  (On a scale of 1=Totally Agree to 5=Totally Disagree, 6= Not applicable/ No opinion.) |
| 8. Space for optional extra comments: …………………………. |
