# Supplementary material for: Effectiveness of oncogenetics training on general practitioners' consultation skills: a randomized controlled trial
Source: Genet Med. 2013 May 30;16(1):45–52. doi: 10.1038/gim.2013.69 (PMC3914027; doi:10.1038/gim.2013.69)
Supplement: Supplementary Table S5 [file gim201369x5.doc]

| **Table S5. Demographics and Practice Characteristics Questionnaire** |
| --- |
| 1. You are:    - Male    - Female |
| 1. Age: …years old |
| 1. Number of years experience as Family Physician: …years |
| 1. In which type of practice do you work?    - Solo practice    - Duo practice    - Group practice    - Community Health Center    - Other |
| 1. Degree of Urbanization of Practice Area:    - Metropolitan area (>100,000 residents)    - City (30,000 – 100,000 residents)    - Small Town (5,000 – 30,000 residents)    - Rural area (<5,000 residents in largest village) |
