# Supplementary material for: Effectiveness of oncogenetics training on general practitioners' consultation skills: a randomized controlled trial
Source: Genet Med. 2013 May 30;16(1):45–52. doi: 10.1038/gim.2013.69 (PMC3914027; doi:10.1038/gim.2013.69)
Supplement: Supplementary Table S6 [file gim201369x6.doc]

| **Table S6. Demographics and Practice Characteristics of Participants (n)** | | | |
| --- | --- | --- | --- |
|  | **Control Group (n=18)** | **Intervention Group (n=38)** | **Chi-square/**  **Mann-Whitney**  **Test**  **(P values)** |
| **Sex** |  |  |  |
| Female | 12 | 29 | .446 |
| Male | 6 | 9 |  |
| **Age in years** |  |  |  |
| 30-39 | 3 | 14 |  |
| 40-49 | 5 | 6 |  |
| 50-59 | 7 | 12 | .318 |
| 60-69 | 2 | 4 |  |
| Unknown | 1 | 2 |  |
| **Professional experience in years** |  |  |  |
| less than 10 | 4 | 9 |  |
| between 10 and 19 | 3 | 7 |  |
| between 20 and 29 | 7 | 6 | .679 |
| between 30 and 39 | 0 | 2 |  |
| between 40 and 49 | 0 | 1 |  |
| Unknown | 4 | 13 |  |
| **Practice Type** |  |  |  |
| Solo practice | 1 | 5 |  |
| Duo practice | 4 | 7 |  |
| Group practice | 2 | 3 | .364 |
| Community Health Center | 5 | 3 |  |
| Other | 4 | 12 |  |
| Unknown | 2 | 8 |  |
| **Practice Setting** |  |  |  |
| Metropolitan area (>100,000 residents) | 10 | 11 |  |
| City (between 30,000 and 100,000 residents) | 1 | 6 |  |
| Small Town (between 5,000 and 30,000 residents) | 3 | 10 | .145 |
| Rural area (<5,000 residents in largest village) | 1 | 0 |  |
| Unknown | 3 | 11 |  |
