# Supplementary material for: Effectiveness of oncogenetics training on general practitioners' consultation skills: a randomized controlled trial
Source: Genet Med. 2013 May 30;16(1):45–52. doi: 10.1038/gim.2013.69 (PMC3914027; doi:10.1038/gim.2013.69)
Supplement: Supplementary Materials and Methods [file gim201369x7.doc]

**Supplemental material**

**Details of background of Methods; Measurements of genetic consultation skills through standardized patients and Regression Analysis**

**METHODS**

**Measurements of genetic consultation skills**

**Standardized Patients**

*Visits*

Twelve experienced SPs (5 male/7 female) were trained by JJR and EJFH to each play one of three oncogenetic cases. All of the four SPs trained to play the breast cancer case were female, while one of the familial colon cancer case SPs and two of the melanoma case SPs were female. They received a comprehensive written account of the clinical scenario and a full briefing of their role. Thereafter, a training session with two of the researchers (EJFH and JJR) clarified the purpose of the project and the standardized roles. The three oncogenetic cases were comparably recognizable and urgent to be able to train all the SPs the first day together on presentation and checklist items. The second training day the SPs were trained separately for each one of the three cases on specific clinical history. In case the GP would indicate to proceed to a physical examination, the SPs handed over to the GP a standardized leaflet summarizing the main PE findings fitting the specific oncogenetic case (see Table S1. Description of Cases presented by Standardized Patients).

At each measurement time (T0, T1 and T2) each GP was visited by one SP. Each SP presented a different case. The GPs were therefore confronted with three different standardized cases, played by three different SPs. The SPs were blinded to whether the GP belonged to the intervention or control group. The GPs were informed an SP would consult during office hours; however they were unaware of exact date and time. After telephoning the office assistant the SP made a standard ten-minute appointment, insisting that their identity would not be disclosed to the GP; however they revealed their identity on entering the GP’s office. At the beginning of each simulated encounter, the SPs gave instructions asking the GPs to conduct the consultation authentically. Immediately after each visit, the SPs completed a uniform, predefined checklist to assess GPs’ genetic consultation skills.

*Cases*

Experts in family medicine, clinical genetics, and education selected the oncogenetic cases and wrote the clinical scenarios (EJFH, JJR, EJvdJ, CvdV) using the following criteria: relatively high prevalence in family practice; diagnostic features identifiable through family history and family tree drawing (Table S1); suitability for a discussion on efficiency considerations (efficient referral to clinical genetics specialist and timeliness of referral); suitability for scoring with one uniform checklist; the presence of important features for physician education; feasibility of realistic SP performance; and coverage of a broad spectrum of oncology cases commonly presented in family practice. [^23^](#_ENREF_23) Using comparable case scenarios according to the criteria mentioned, enabled comparing potential changes in checklist scores between the three different measurement times.

*Checklist*

The research team developed a 28-item checklist to quantify evidence judged necessary to assess GPs’ genetics consultation skills reflecting training outcomes’ covering the full scope of good practice consultations: key ingredients related to family history taking, genetic risk assessment, and referral to genetics specialists. To validate the checklist, three experts in family medicine education and four clinical geneticists evaluated the applicability of the items and cases (Table S2). Items judged not suitable for scoring (i.e. less than 75% of experts agreed on inclusion) were removed. Items were rated as “yes, observed” (score=1), “no, not observed at all” (score=0), or “?, unclear” (not scored = missing). The scores were summed and the proportion of the maximum possible score (1 for each item, ignoring the missing items[^24^](#_ENREF_24)) was determined.

*Training of standardized patients and finalization of checklist*

One month before the first office visit, 12 SPs were trained, in groups of four, to play the three cases (one case consistently per group of 4) and score the checklist on GP’s performance consistently. Training took 16 hours spread over two days. Realistic portrayal was promoted by SPs practicing ten-minute consultations with ten real GPs (not training participants) in a studio in a GP practice setting. Training focused particularly on the use of introductory phrases and on helping them avoid giving away clues as to their role. SPs were also trained to fill in the checklist, to be completed immediately after a consultation during the trial, and their colleague SPs practiced their same role. Their checklist scores were compared with those of the SP trainers (EJFH and JJR), the supposed “gold standard”. The practice-consultations were videotaped, scored, and judged to have sufficient face validity for each SP by a panel of four GPs who did not participate in these training sessions and were not otherwise involved. Differences in rating between the panel members were discussed and final alterations made to the measurement scale and checklist. The panel and the SPs independently re-rated the transcripts using the modified checklist. Agreement between SPs and panel ranged from 87% to 98%, with Cohen’s kappa 0.87, which was deemed satisfactory for commence of the practice visits.

**Regression Analysis**

Regression analysis was used to investigate improvement of genetic consultation skills immediately after the course using the checklist scores at T1 (ScoreT1) as dependent variable and the checklist scores at T0 (ScoreT0) and the indicator variable Train (0: control group; 1: intervention group) as independent variables. To improve interpretation and numerical stability, the independent variable ScoreT0 was centered and the resulting variable ScoreT0, equal to ScoreT0-Mean (ScoreT0) was used in the analysis.

Each participant was rated on one of the three SP cases at T0, T1, and T2. As the roles might vary in difficulty and the case order varied between participants, we corrected for these differences by extending the regression equation with four terms corresponding to four indicator variables (values: 0, 1), the dummy variables D_1_-D_4_. This suffices to represent the effects of the five possible different role pairs in comparisons at T1 and T0. Role pair 1 is indicated by (D_1_=1, D_2_=D_3_=D_4_=0), role pair 2 by (D_2_=1, D_1_=D_3_=D_4_=0), etcetera, and role pair 5, is indicated by (D_1_=D_2_=D_3_=D_4_=0). Thus, the effect of the intervention was assessed using the model:


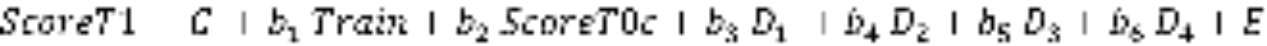


In the model C (Constant) represents the intercept of the regression equation, i.e. the predicted score at T1 for a participant in the control group (Train=0) with a score at T0 equal to the mean score (ScoreT0=0), and being visited by role pair 5 (D_1_=D_2_=D_3_=D_4_=0). Regression coefficient b_1_ represents the effect of the intervention (Train=1), i.e. the increase of the score at T0 due to a participant being a member of the intervention group. Coefficient b_2_ is the effect of the pretest (ScoreT0), b_2_ ScoreT0 being the part of the score at T1 that can be predicted from the score at T0. Regression coefficients b_3_-b_6_ indicate the increase of the score at T1 when a participant was visited by role pair 1, 2, 3, or 4, respectively, instead of being visited by the reference role pair (role pair 5). Term E (Error) is the part of the score at T1 that cannot be explained by the predictors in the regression model (the residual).

In a similar procedure, using ScoreT2 as dependent variable, retention of knowledge was analyzed.

The regression coefficient b_1_ corresponding to Train represents the net gain in performance (expressed as proportion of the maximum score) due to the intervention. The corresponding standardized regression coefficient indicates the effect size. According to Cohen’s categorization (Cohen, 1988) values 0.1, 0.3, and 0.5 indicate small, moderate, and large effect sizes, respectively.

The mean checklist scores and corresponding 95% confidence intervals were calculated for the two groups at T0, T1, and T2. Because the raw means are not corrected for varying role orders, the differences of the raw means may differ considerably from the intervention effect found in the regression analysis. It is important to note that the intervention effect inferred from the raw means is biased, whereas the regression analysis provides an unbiased estimator.

Satisfaction with the intervention was analyzed by calculating mean scores, 95% confidence intervals, and standard deviations for the pooled data from the satisfaction questionnaire. The data from the applicability questionnaire were analyzed in the same way. All analyses were performed using SPSS version 19 (SPSS, Chicago, IL).
